# Cortical Components of Reaction-Time during Perceptual Decisions in Humans

**DOI:** 10.1371/journal.pone.0143339

**Published:** 2015-11-23

**Authors:** Jacek P. Dmochowski, Anthony M. Norcia

**Affiliations:** Department of Psychology, Stanford University, Stanford, CA, United States of America; Università di Trento, ITALY

## Abstract

The mechanisms of perceptual decision-making are frequently studied through measurements of reaction time (RT). Classical sequential-sampling models (SSMs) of decision-making posit RT as the sum of non-overlapping sensory, evidence accumulation, and motor delays. In contrast, recent empirical evidence hints at a continuous-flow paradigm in which multiple motor plans evolve concurrently with the accumulation of sensory evidence. Here we employ a trial-to-trial reliability-based component analysis of encephalographic data acquired during a random-dot motion task to directly image continuous flow in the human brain. We identify three topographically distinct neural sources whose dynamics exhibit contemporaneous ramping to time-of-response, with the rate and duration of ramping discriminating fast and slow responses. Only one of these sources, a parietal component, exhibits dependence on strength-of-evidence. The remaining two components possess topographies consistent with origins in the motor system, and their covariation with RT overlaps in time with the evidence accumulation process. After fitting the behavioral data to a popular SSM, we find that the model decision variable is more closely matched to the combined activity of the three components than to their individual activity. Our results emphasize the role of motor variability in shaping RT distributions on perceptual decision tasks, suggesting that physiologically plausible computational accounts of perceptual decision-making must model the concurrent nature of evidence accumulation and motor planning.

## Introduction

Behavioral analyses of perceptual decision-making have been firmly grounded in the theoretical framework provided by sequential sampling models (SSMs) [1], whose hallmark is the *decision variable* (DV), an abstract entity quantifying the amount of evidence favoring one alternative versus the other [2, 3]. The temporal evolution of the DV determines behavioral outcomes, such that SSMs make concrete predictions about both the accuracy and reaction time (RT) of a perceptual decision. It is widely hypothesized that RT may be decomposed into at least three independent sources: the time required to encode the stimulus, the time required to accumulate sufficient evidence for commitment to a choice, and the time required to then execute the corresponding action. In the drift-diffusion model (DDM), a highly-influential instantiation of the SSM, these three stages are sequential and non-overlapping, such that their durations add to form the total RT [4].

The pursuit of the neural basis of perceptual decisions has been marked by attempts to match neural signals (for example, firing rates of single neurons or mass field potentials) to model-generated DVs [2, 3]. These efforts have focused on identifying the neural correlates of the evidence accumulation process. The contribution of the motor system to observed RT has been accounted for by including an additive delay to the back-end of the decision process, in order to model static delays such as those expected from corticospinal activity. In contrast, increasing evidence suggests that decision formation is gated through the motor system in a concurrent fashion [5–10], and as such, activity in the motor system has been found to mimic the DV [11–14].

An alternative view to that proposed by SSMs is “continuous flow”, in which evidence accumulation and motor planning of multiple candidate actions occurs in parallel [15–17]. Here we provide neural evidence for the continuous-flow model during perceptual decisions in human. We recorded scalp electroencephalography (EEG) during a random-dot motion task, and employed a novel component analysis to decompose the data into three temporally uncorrelated sources that are reliably evoked by the experimental paradigm. Each of these sources shows ramping activity that peaks at or near the time of response, with steeper and shorter ramps indexing fast RTs. While the activity of one of these sources, likely arising in parietal cortex, is modulated by task difficulty, the other two are independent of strength-of-evidence and have topographies consistent with sources in the premotor and motor cortex, respectively. Their covariation with RT is then shown to temporally overlap with the accumulation of evidence. Finally, we fit the behavioral data to a popular SSM and show that the resulting DV is better explained by the combined activity of the three components than by any individual one, including the parietal component.

Our findings cast doubt on a sequential view of decision-making in which evidence accumulation precedes motor planning in a discrete fashion, instead lending support to the continuous flow view of decision-making. We provide direct evidence of RT variability in the motor system, and suggest that the abstract DV may translate to concurrent processes operating across multiple, spatially distributed cortical areas.

## Methods

### Experimental paradigm

Data were collected from 28 participants (11 females, ranging in age from 18 to 62 years with a mean of 26 years) with normal or corrected-to-normal visual acuity. Written informed consent was obtained prior to study initiation under a protocol that was approved by the Institutional Review Board of Stanford University. Subjects performed a fine motion-direction discrimination task comprised of 3-4 blocks of 70 trials each: 23 (5) of the 28 subjects performed 3 (4) blocks, depending on their self-reported ability to stay attentive and alert throughout the experiment. A dynamic random-dot stimulus was presented using in-house software on a contrast linearized CRT monitor (HP P1230) at a resolution of 1600-by-1200 pixels and a vertical refresh rate of 60 Hz. The stimulus subtended a visual angle of 4.8 degrees. Subjects were instructed to center fixation onto a cross prior to trial onset and maintain fixation throughout the trial. Each trial began with one second of Brownian motion (“boiling”), consisting of an independent random-walk at each dot, with dot positions updated at rate of 20 Hz. The boiling phase was followed by one second of coherent motion in which the dots followed a dominant motion-direction. The coherence bandwidth (i.e., the sector angle from which direction vectors are sampled during the coherent motion period) was set to 30 degrees for all conditions, with conditions differing on the mean direction relative to vertical motion: seven conditions linearly ranging from 79.8 to 100.2 degrees in increments of 3.4 degrees were employed. This led to three difficulty levels: “easy” (±10.2 degree mean deviation), “medium” (±6.8 degree mean deviation), “hard” (±3.4 degree mean deviation), in addition to an ambiguous vertical condition (0 degree mean deviation). Subjects were asked to indicate, with the press of one of two buttons, whether they perceived the dots to be moving counter-clockwise (“left”) or clockwise (“right”) of vertical, and were instructed to respond as quickly and accurately as possible. Responses to the left (right) were made by the left (right) hand. The valid response period comprised of the duration of coherent motion, and trials during which RT exceeded 1000 ms were marked as lapses. The inter-trial interval was fixed to 3s.

### Data pre-processing

The EEG was acquired using a 128-channel electrode array (Electrical Geodesics Inc, OR) at a sampling rate of 500Hz with a vertex reference electrode. Initial pre-processing was performed using in-house software. Signals were band-pass filtered between 0.3 and 50 Hz. Channels in which 15% of the samples exceeded a fixed threshold of 30 *μV* were replaced with a spatial average of the six nearest neighbors. Within each trial, channels containing samples exceeding 30 *μV* were rejected. Trials containing at least such 7 channels were rejected altogether. This resulted in an average of 16%±13% of rejected data points per subject, treated as missing data for subsequent analysis. The EEG was then re-referenced to the common average of all channels. Data were epoched to retain only the peri-trial interval, yielding 2 second data records which were then imported into the MATLAB environment in which all subsequent processing was performed. This included regressing out the horizontal and vertical electrooculogram (EOG) channels, notch-filtering of the 20 Hz dot-update component and its 40 Hz harmonic, and baselining to the first sample of the boil period. For each trial, we formed a stimulus-locked record (spanning one second beginning at the onset of coherent motion) as well as a response-locked record beginning 750 ms before and ending 250 ms after the time of the button press.

### Reliable components analysis

In order to identify the cortical sources recruited by the perceptual decision task, we performed a component analysis rooted in the maximization of trial-to-trial reliability. Reproducibility of evoked responses has been recently proposed as a novel criterion by which to perform dimensionality reduction of continuous neural responses to naturalistic stimuli [18, 19] and steady-state visual evoked potentials [20]. Here, we apply the reliability criterion to the context of conventional time-domain evoked responses, extracting a set of sources capturing the reliable activity elicited by the experimental paradigm. In contrast to principal components analysis (PCA) which forms components according to a criterion of variance explained, “Reliable Components Analysis” (RCA) projects the data onto a space in which the trial-to-trial covariance of the projected activity is maximized. The technique thus exploits the fundamental assumption underlying evoked responses, namely that the signal-of-interest is spatiotemporally reproducible across trials. Below we describe the general algorithm, with specifics of the implementation found in the next subsection.

Let **X**_*n*_ denote the space-by-time data matrix of neural activity observed during experimental trial *n*. The input into RCA is the set of all *N* such congruent data matrices {**X**_1_, …, **X**_*N*_}, where *N* is the total number of trials. In order to maximize covariance across trials, we form the following trial-aggregated data matrices:

$$\begin{array}{ccc} {\bar{X}}_{1} & = & \left[\begin{array}{cccc} X_{p_{1}} & X_{p_{2}} & \ldots & X_{p_{P}} \end{array}\right],\\ {\bar{X}}_{2} & = & \left[\begin{array}{cccc} X_{q_{1}} & X_{q_{2}} & \ldots & X_{q_{P}} \end{array}\right], \end{array}$$ (1)

where $P_{i}=\left\{\left(p_{i},q_{i}\right)\right\}=\left\{\left(1,2\right),\left(1,3\right),\ldots ,\left(N-1,N\right),\left(N,N-1\right),\ldots ,\left(3,1\right),\left(2,1\right)\right\}$ denotes the set of all *P* = *N* × (*N* − 1) order-dependent unique trial pairs. In a 3-trial experiment, $P_{i}=\{(1,2),(1,3),(2,3),(3,2),(3,1),(2,1)\}$, such that *p*_*i*_ = {1, 1, 2, 3, 3, 2}, *q*_*i*_ = {2, 3, 3, 2, 1, 1}, and *P* = 6.

A common spatial filter **w** is then applied to ${\bar{X}}_{1}$ and ${\bar{X}}_{2}$, yielding

$$\begin{array}{ccc} {\bar{y}}_{1} & = & {\bar{X}}_{1}^{T}w,\\ {\bar{y}}_{2} & = & {\bar{X}}_{2}^{T}w, \end{array}$$ (2)

where ^*T*^ denotes matrix transposition. The correlation coefficient between the filtered data records is given by:

$$\begin{array}{c} \bar{\rho }=\frac{{\bar{y}}_{1}^{T}{\bar{y}}_{2}}{{\left({\bar{y}}_{1}^{T}{\bar{y}}_{1}\right)}^{1/2}{\left({\bar{y}}_{2}^{T}{\bar{y}}_{2}\right)}^{1/2}}. \end{array}$$ (3)

Substituting Eq (2) into Eq (3) yields:

$$\begin{array}{c} \bar{\rho }=\frac{w^{T}R_{12}w}{{\left(w^{T}R_{11}w\right)}^{1/2}{\left(w^{T}R_{22}w\right)}^{1/2}}=\frac{w^{T}R_{12}w}{w^{T}R_{11}w}, \end{array}$$ (4)

where

$$\begin{array}{ccc} R_{11} & = & \frac{1}{2TP}\sum_{i=1}^{2TP}X_{p_{i}}X_{p_{i}}^{T}=\frac{1}{2TP}\sum_{i=1}^{2TP}X_{q_{i}}X_{q_{i}}^{T}=R_{22},\\ R_{12} & = & \frac{1}{2TP}\sum_{i=1}^{2TP}X_{p_{i}}X_{q_{i}}^{T}, \end{array}$$ (5)

where *T* is the number of temporal samples per trial, **R**_11_ and **R**_22_ denote the within-trial spatial covariance matrix (their equivalence follows from the construction of ${\bar{X}}_{1}$ and ${\bar{X}}_{2}$), and **R**_12_ is the *across-trial* spatial covariance matrix. The optimization aims to find the spatial filter **w** which maximizes the ratio of across- to within-trial covariance:

$$\begin{array}{c} \arg\max_{w}\bar{\rho }. \end{array}$$ (6)

The solution to Eq (6) takes the form of a conventional eigenvalue problem:

$$\begin{array}{c} {\bar{\rho }}^{*}R_{11}w^{*}=R_{12}w^{*}, \end{array}$$ (7)

where ${\bar{\rho }}^{*}$ is the eigenvalue corresponding to the maximal correlation coefficient achieved by projecting the data onto the spatial filter $w^{*}$. There are *D* such solutions, ranked in decreasing order of trial-to-trial reliability: ${\bar{\rho }}_{1}^{*}>{\bar{\rho }}_{2}^{*}>\ldots >{\bar{\rho }}_{D}^{*}$, where $D=\text{min}\;[\text{rank}\;(R_{11}),\text{rank}\;(R_{12})]$. Unlike PCA, the associated eigenvectors, $w_{1}^{*},w_{2}{,}^{*}\ldots ,w_{D}^{*}$ are *not* generally orthogonal. On the other hand, the trial-aggregated component waveforms recovered by the multiple eigenvectors are mutually uncorrelated.

### Implementation of RCA

Data from 3 of the 24 subjects were excluded from all analyses due to their task performance not deviating significantly from chance level (50% accuracy). Data from a fourth subject was excluded due to the observed accuracy falling systematically *below* chance. From the remaining participants, only trials in which a correct response was received within the 1-second coherent motion period were considered for learning the reliable components (RCs). To maximize the sensitivity of RCA to recover low signal-to-noise ratio (SNR) sources, for each hand we pooled response-locked data records across subjects and conditions. RCA was then performed separately for left- and right-responses. When computing the eigenvalues of Eq (7), we regularized the within-trial pooled covariance by keeping only the first *K* dimensions, where *K* = 9 corresponded to the “knee” of the eigenvalue spectrum, in the spectral representation of **R**_11_.

Averaged across left- and right-responses, the (descending) eigenvalues corresponding to the first 5 RCs were: 0.075, 0.013, 0.0065, 0.0025, 0.0014. Consequently, we chose to truncate the RC space after the first three components. To analyze the spatial topographies of these three RCs, we examined not the spatial filter weights but rather the scalp projection of the activity recovered by the filters. This projection is generally more informative than the weights in that it encompasses both the filter weights as well as the data that is being multiplied by them [21]. Specifically, let **W** denote a matrix whose columns represent the weight vectors **w** yielded by RCA. The projections of the recovered sources onto the sensor data are given by [21, 22]:

$$\begin{array}{c} A=R_{11}W{\left(W^{T}R_{11}W\right)}^{-1}. \end{array}$$ (8)

The columns of **A** represent the pattern of electric potentials that would be observed on the scalp if only the source signal recovered by **w** was active, and inform us of the approximate location of the underlying neuronal sources.

To examine the temporal dynamics of the three RCs, we projected the sensor data from all leftward (rightward) responses onto the three spatial filters maximizing reliability over all left-response (right-response) trials. blackIn order to determine whether the RC amplitude at each time instant explained a statistically significant amount of RT variance, we performed a permutation test which scrambled the RTs across trials. The p-value followed as the proportion of 500 permutations whose resulting *R* ^2^ value exceeded the true (i.e., unpermuted) explained variance. The p-values were then corrected for multiple comparisons using the false discovery rate [23]. Note that to analyze the effect of errors on RC dynamics, we projected the activity of error trials onto the spatial filter corresponding to the hand used to make the response. To relate the slopes of the observed RC ramps to individual differences in mean RT, we performed linear regression over the 200 ms leading up to the button-press for RCs 1 and 2, and during the interval (−260, −100) ms for RC3. These ranges were chosen by inspecting the ramps of grand mean time courses for each RC.

To compute the centroparietal positivity (CPP) [12, 24] and lateralized readiness potential (LRP) [25–27], earlier decision-making components which were contrasted with the RCs, we followed the procedure of [24]. Scalp data were first transformed using the current source density (CSD) method of [28] by way of the CSD toolbox [29]. The CPP was then constructed by averaging the activity of the two electrodes closest to standard location CPz. To compute the LRP, we subtracted the activity of the ipsilateral electrode closest to site FC3/FC4 from the activity of the contralateral electrode closest to site FC4/FC3, depending on whether the left or right hand was used to make the button-press. To enable a spatial comparison between the CPP/LRP and the RCs, we computed the scalp projections of the CPP and LRP in a manner analogous to Eq (8). These scalp projections were computed as the temporal correlation coefficient between the CPP/LRP time series and the raw signal at each electrode (pooled across all subjects and trials). Note that this procedure is mathematically equivalent to Eq (8), with the resulting topographies reflecting the activity observed if only the sources of the CPP/LRP were active. To compare the temporal dynamics of the RCs with those of the CPP/LRP, we computed the conditional (i.e., fast versus slow RT, easy versus hard) response-locked means across all valid and correct responses.

### Computational modeling of behavior

To model the behavior observed in our perceptual decision task, we employed the Ornstein-Uhlenbeck (OU) SSM which models a DV according to [1]:

$$\begin{array}{c} dx=(\lambda x+k)dt+\sigma dw, \end{array}$$ (9)

where *x* is the accumulated evidence favoring one alternative over the other, *λ* is a leak-strength (*λ* < 1) or urgency (*λ* > 1) parameter, *k* is the drift rate, and *σdw* is the zero-mean Wiener noise which has a standard deviation of $\sigma \sqrt{dt}$, where *dt* is the time increment of the process. The starting point of the process was *x* = 0, with a correct (erroneous) decision made when *x* ≥ 1 (*x* ≤ −1). The OU model reduces to the standard drift diffusion model (DDM) with *λ* = 0 [1].

We sought to identify the model parameters yielding an RT distribution matching our behavioral data. To that end, we performed a coarse grid search, similar [14], over the following values: *λ* ∈ {−9, −8.8, …, 8.8, 9}, *k* ∈ {0, 0.02, …, 0.3}, *σ* ∈ {0.02, 0.04, …, 1.5}. Additionally, a fourth free parameter representing the “non-decision time” *τ*_*o*_ ∈ {0, 0.05, …, 0.3}, accounting for fixed latencies due to sensory encoding and corticospinal delays was included in the grid search. The objective function to be minimized was defined by:

$$\begin{array}{c} \sum_{i=0}^{3}\text{KS}\left({\text{RT}}_{\text{model}}^{i},{\text{RT}}_{\text{data}}^{i}\right), \end{array}$$ (10)

where KS is the Kolmogorov-Smirnov (KS) statistic measuring the distance between empirical and model distributions, ${\text{RT}}_{\text{model}}^{i}$ is the RT distribution predicted by the model for difficulty index *i*, and ${\text{RT}}_{\text{data}}^{i}$ is the empirical distribution of observed RTs at difficulty *i*.

To learn a single model which explains behavior on all difficulty levels, we performed a joint optimization in which the drift rate on the medium, hard, and vertical conditions is linearly related to the base (easy) drift rate: *k*(easy) = *k*, *k*(medium) = *α*_1_ *k*, *k*(hard) = *α*_2_ *k*, *k*(vertical) = 0. To estimate the appropriate weighting scalars *α*_1_ and *α*_2_, we employed the expression relating mean RT to drift rate for the pure DDM [30]. Iterating the noise variance parameter *σ* to fit the mean RTs for all conditions led to: *α*_1_ = 0.56 and *α*_2_ = 0.87. These scalars were then used in Eq (10) above to fit the base drift rate *k* and other free parameters. In order to jointly fit error and correct RTs, we inverted the sign of all incorrect RTs in both model and empirical distributions, effectively using the negative part of the distribution to hold the error RTs (Rafael Polania, personal communication). For each difficulty level, empirical RT distributions were constructed by pooling RTs across subjects.

The minimum value of the aggregated KS objective function Eq (10) was 0.24. We performed a chi-squared goodness-of-fit test to evaluate the quality of the fit between the resulting model and empirical RT distributions using 8 equally spaced quantiles (ignoring error trials which were sparse for all but the hard condition) [31]. The resulting chi-squared values for the easy, medium, hard, and vertical conditions were 3.53, 7.62, 9.27, and 4.48, respectively, corresponding to p-values of 0.83, 0.37, 0.23, and 0.72, respectively. Thus, the null hypothesis of the empirical and model distributions not differing could not be rejected for any of the conditions.

The parameter values minimizing the objective function were used to generate *N* = 10000 realizations of a model DV. In order to regress the mean RC time courses onto this DV, we aggregated both model and RC data across difficulties, effectively learning the mapping between RCs and DV jointly (i.e., so as not to have a separate mapping for each difficulty). To adjust *R* ^2^ values for additional degrees-of-freedom, we used the following correction: *R* ^2^: = 1 − (1 − *R* ^2^)(*n* − 1)/(*n* − *p* − 1) where *n* is the number of samples being predicted and *p* is the number of predictors [32].

## Results

**Fig 1:**
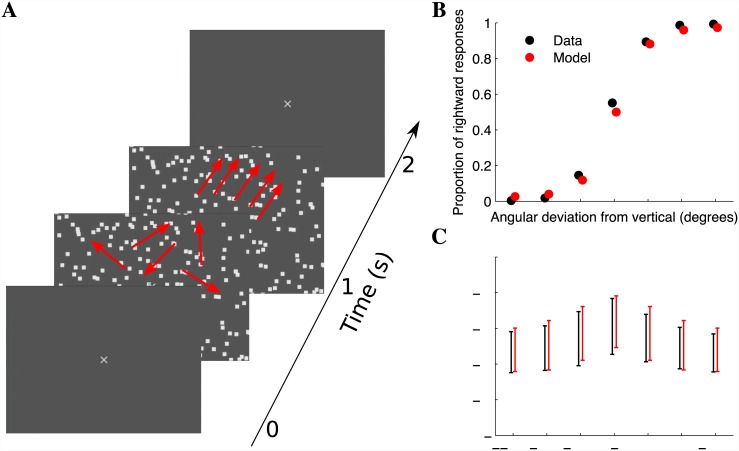
Behavioral performance on a motion-direction discrimination task in which subjects rapidly report the perceived direction of motion (counter-clockwise or clockwise from vertical). **(A)** Trials commence with a 1 second preparatory period consisting of a Brownian (incoherent) motion stimulus, followed by 1 second of coherent motion which marks the valid response window. **(B)** Proportion of rightward (clockwise) responses as a function of the angular deviation from vertical (positive values indicate clockwise direction). Resulting accuracies are 99%, 98%, and 87% on the easy, moderate, and difficult discriminations, respectively (averaged across clockwise and counter-clockwise motion). A SSM was fit to the behavioral data, and the predictions corresponding to the optimal fit are shown in red with a small horizontal offset. **(C)** Reaction time (RT) distribution as a function of the angular deviation from vertical. Mean RTs vary from 464 ms on the easy discriminations to 611 ms for vertical motion. Error bars indicate the standard deviation across *N* = 810 trials per condition, and the RT predictions of the optimized SSM are indicated in red (*N* = 810 model realizations per condition were simulated to construct the error bars).

Accuracy on the random-dot motion task ranged from an average of 99% on easy discriminations (10.2° deviation from vertical) to 87% on difficult conditions (3.4° from vertical), with mean RTs varying from 464 ms on the easy discriminations to 611 ms for (ambiguous) vertical motion (Fig 1). A computational model of behavior on the decision-making task was constructed by finding the OU model parameters that generated RT distributions most consistent with the empirical RTs (see Methods). The model RTs and accuracies corresponding to the optimal model fits are shown in red, indicating that the SSM generated a DV predicting both RT and accuracy across conditions.

Evoked responses from 128 scalp electrodes were obtained from all subjects as they performed the task. From the sensor-space data, we sought to identify a small set of cortical sources whose activity captures task-processing. We thus employed the recently proposed RCA method, a dimensionality reduction technique akin to principal components analysis (PCA), differing in that the criterion used to select the components is trial-to-trial reliability as opposed to explained variance [18–20]. RCA projects the sensor data onto a low-dimensional space in which trial-to-trial reliability is maximal, yielding component time courses and scalp topographies corresponding to the underlying neural sources. We performed the analysis separately for responses to the left and right, and retained three RCs for subsequent analysis (the number of retained components followed by inspection of the eigenvalue spectrum; see Methods for details). To gain insight into the processes reflected by the identified components, we then analyzed the following: (1) spatial (scalp) topography and the presence of lateralization with response-hand, (2) effect of RT on temporal dynamics, (3) effect of strength-of-evidence on temporal dynamics, and (4) the temporal dynamics on error trials.

### Reliable components covary with reaction time

**Fig 2:**
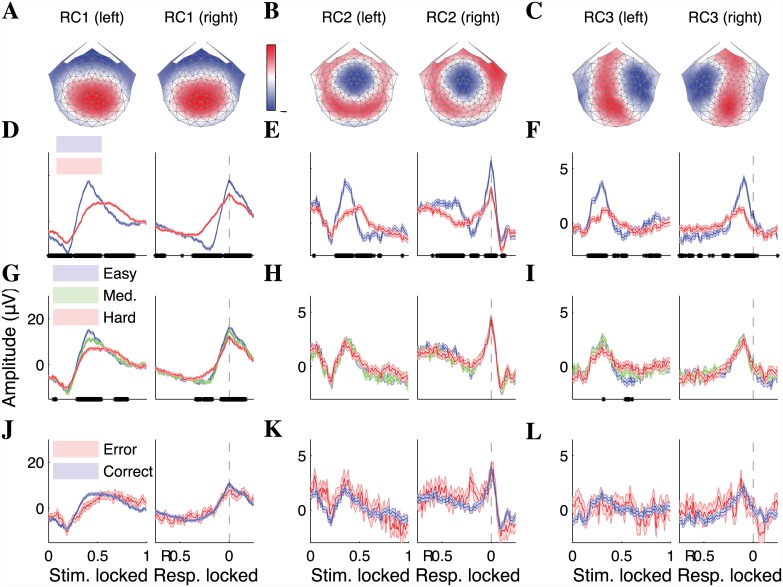
Three topographically distinct components covary with reaction time (RT) on a motion-direction discrimination task. Spatial topographies of (**A**) RC1 and (**B**) RC2 exhibit poles over medial parietal and frontocentral cortex, respectively, and are independent of response-hand. (**C**) The topography of RC3 contralateralizes with the hand used to indicate the response. (**D**)–(**F**) The temporal dynamics of all three RCs peak earlier and with higher amplitude on fast RT trials (stimulus-locked panels on left). Moreover, their time courses peak at or near the time-of-response and discriminate fast and slow responses throughout the trial (response-locked panels on right; statistical significance defined by two-sided Wilcoxon rank-sum test, corrected for multiple comparisons using the FDR, *p* < 0.05, indicated by asterisks along the horizontal axis). (**G**)–(**I**) Despite all three components covarying with RT, only RC1 is dependent on difficulty, with activity on easy trials peaking earlier and higher than during hard trials. (**J**)–(**L**) All three components fail to show covariation with choice accuracy. In all curves, shaded error bars denote the standard error-of-the-mean (SEM).

The spatial topographies of the first two components, RC1 and RC2, exhibit maxima over medial parietal and medial frontocentral cortex, respectively (Fig 2A and 2B) and lack lateralization with response-hand. On the other hand, RC3 is marked by a contralateralized topography, with the zero-potential contour (white) separating maxima of opposite polarity lying left (right) of the vertex for responses to the right (left) (Fig 2C). Note that these RCs were computed from data pooled across subjects and conditions (see Methods). We also separately performed RCA on data from individual participants and found that the parietal topography of RC1 is recovered for all subjects; on the other hand, RC2 and RC3 are lower SNR components that require aggregated data to be extracted (data not shown).

To examine the effect of RT on the temporal dynamics of the RCs, we split trials into fast and slow responses (less or greater than the median RT), and plotted the resulting time courses locked to the onset of coherent motion (left panels of Fig 2D–2F). For all three RCs, the temporal dynamics during fast trials peaked earlier and with higher amplitude than that during the slow trials. As a result, activity of all three RCs significantly discriminated fast and slow responses throughout extended portions of stimulus-locked time, indicated by asterisks on the horizontal axis (Wilcoxon rank sum test, *N*_fast_ = 1814, *N*_slow_ = 2051, corrected for multiple comparisons using the false discovery rate [FDR], *p* < 0.05). For RC1, there was also a pronounced effect immediately at coherent motion onset, with significantly lower initial amplitudes observed during fast trials (Fig 2D, time 0). RC1 activity decreased during the boil period, with a deeper negative progression during trials that would eventually have fast responses (responses were baselined to the onset of the boil period).

The later portion of stimulus-locked activity is a mixture of sensory and decision signals. In order to more closely tie the evoked activity to the decision process, we also performed a response-locked analysis in which time courses were locked to the button-press. Viewed in this temporal reference, the dynamics of all three RCs peaked at (i.e., RCs 1 and 2) or shortly before (i.e., RC3) the time of response (right panels of Fig 2D–2F). The RCs exhibit “ramping” leading up to the peak of the curve, with larger slopes and higher peaks during fast-response trials. Here and throughout the paper, the peaking of activity near the time of the button press was *not* imposed by the analysis. As was the case with stimulus-locked responses, response locked curves of all three RCs significantly discriminate fast and slow trials across a broad temporal range. It is also noteworthy to point out that the modulation of temporal dynamics by RT was apparent even *after* the button-press for RCs 1 and 2, hinting at an influence of decision uncertainty on RC dynamics (see Discussion).

While all three RCs were modulated by speed-of-response, only RC1 showed differentiated activity when splitting the trials according to the strength-of-evidence (Fig 2C). Hard trials were marked by longer, shallower ramping activity which peaked at a lower level than that of the easy trials. Statistically significant deviation between easy and hard trials was observed before, during, and after the button press (Wilcoxon rank sum test, *N*_easy_ = 1544, *N*_hard_ = 1323, corrected for multiple comparisons using the FDR, *p* < 0.05). Importantly, no significant differences between easy and hard trials were observed in the dynamics of either RC2 or RC3. Thus, while RC2 and RC3 were modulated by RT, this modulation was not explained by the effect of strength-of-evidence.

To investigate the effect of incorrect choices on temporal dynamics, we examined RC time courses as a function of accuracy, considering only trials from the hard category due to the low number of errors observed in the easy and medium conditions. For all three RCs, choice accuracy had no effect on the observed dynamics (Fig 2D; Wilcoxon rank sum test, *N*_correct_ = 1323, *N*_error_ = 191, corrected for multiple comparisons using the FDR, *p* < 0.05). We expand on the implications of this result in the Discussion.

**Fig 3:**
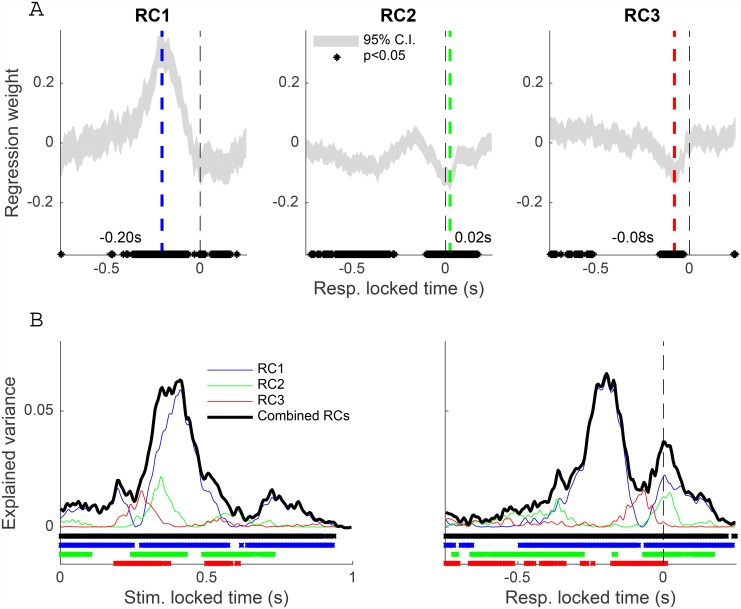
The covariation of the three RCs with RT is contemporaneous and complementary. (**A**) Linear regression weight relating single-trial RC1 amplitude to resulting RT shows statistical significance throughout a broad portion of response-locked time, peaking 200 ms before button-press (statistical significance indicated by the 95% confidence interval of the regression weight excluding zero, shown with asterisks on the horizontal axis). (**B**) Single-trial RC2 amplitude predicts RT before and after the button press, with peak covariation 20 ms post-response. (**C**) The amplitude of RC3 significantly covaries with RT across a wide range leading up to the response and shows a minimum 70 ms before the button-press. (**D**) The proportion of RT variance explained as a function of both stimulus-locked (left panel) and response-locked (right panel) time for each RC. By combining the amplitudes of the three RCs into a multivariate linear regression, a more accurate prediction of RT is obtained (thick black line). Statistically significant explained variance, as computed by a permutation test, is indicated with asterisks above the horizontal axes.

In order to probe the timing of the trial-by-trial relationship between RC dynamics and RT, we regressed single-trial RC amplitude at each time instant (one regression weight + offset) onto the subsequent RT for each component. Statistically significant trial-by-trial predictability, indicated by the 95% confidence interval of the regression weight excluding zero, was found across broad and highly-overlapping temporal windows for all three RCs (Fig 3A). Additionally, we performed a multivariate regression using the instantaneous values of the three RCs and found an increase in the proportion of RT variance explained over that provided by the univariate regressions (Fig 3B). These results suggest that RT follows from the activity of distinct but contemporaneous cortical sources that carry complementary information.

**Fig 4:**
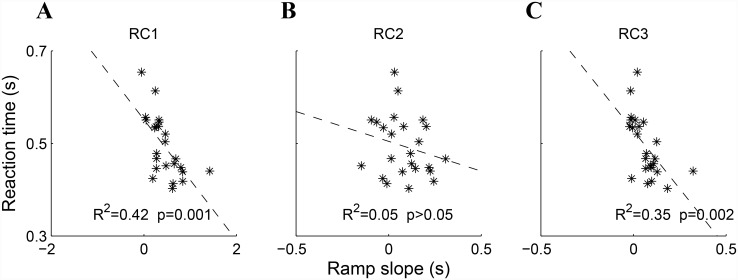
RC ramp slopes predict individual differences in RT. (**A**)The slope of the ascending portion of the mean RC1 time course explains 42% of across-subject variability in mean RT, with steeper gradients indicating shorter RTs (*r* = −0.65, *p* = 0.0007, *N* = 24, p-value computed using the Fisher test). (**B**) No significant covariation with RT is observed for RC2. (**C**) Mean RC3 ramp duration explains 36% of the across-subject variability in mean RT (*r* = 0.60, *p* = 0.002).

Given the possible relationship between ramping neural activity and evidence accumulation, we sought to determine whether the slopes of the observed RC ramps are predictive of subsequent RT. The low SNR of RCs 2 and 3 precluded us from testing this on a single-trial level. Therefore, for each subject we estimated the slope of the ascending part of their mean RC time series and correlated the resulting values with mean RT (see Methods). Significant covariation between ramp slope and RT was observed for both RC1 (Fig 4A; *r* = 0.65, *p* = 0.0007, *N* = 24, p-value computed using the Fisher test) and RC3 (Fig 4C; *r* = 0.59, *p* = 0.002). Moreover, the ramp slopes of RC1 and RC3 are themselves significantly correlated across subjects (*r* = 0.85, *p* < 0.0001).

### Combined activity matches model prediction

**Fig 5:**
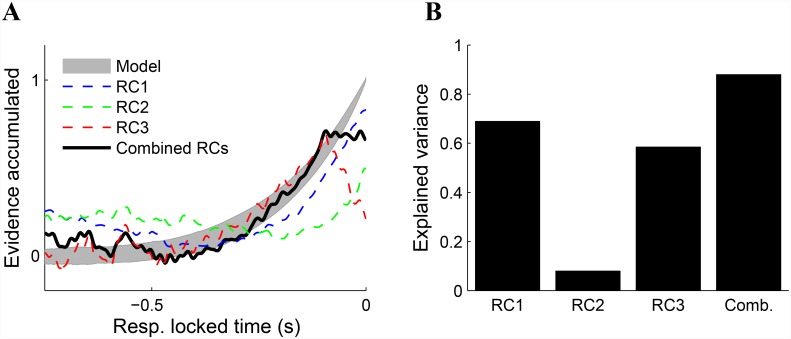
Combined activity of RCs matches prediction of SSM. **(A)** Model DV as generated by realizations of an OU process fit to the behavioral data (grey area denotes ± 1 standard deviation around the mean). Linearly regressing the individual RC activity onto the model yields moderate fits for RC1 and RC3 (blue and red dashed lines, respectively) and a weak fit for RC2 (green dashed line). Solid black line indicates the result of regressing the combined activity of the three RCs onto the model DV. **(B)** RC1, RC2, and RC3 account for 69%, 8%, and 59% of the variance in the model DV, respectively. After accounting for the increase in explanatory variables, the combined activity of the three RCs explains 88% of the model signal.

In order to connect the dynamics exhibited by a behaviorally derived DV to the neural activity of the RCs, a commonly used SSM (the OU process, see Methods) was used to fit the behavioral data from the random-dot motion task. From the optimal fit parameters, a model DV was constructed and 10,000 realizations were simulated. The response-locked temporal evolution of this model DV is shown in Fig 5A (grey area denotes ±1 standard deviation around the mean). We sought to relate this behavior-driven DV to the neural activity of the RCs.

We first linearly regressed the activity of each individual RC onto the DV, effectively scaling and offsetting the components to bring them onto the range of the model. Good fits were obtained for both RC1 and RC3, which explain 69% and 59% of the variability in the model DV, respectively (Fig 5A).

Given that the evolution of the model DV determines RT, and that the activity of all three RCs was modulated by RT, we hypothesized that the DV may be best explained by the combined activity of the three components. To test this, we performed a multivariate regression from the three-dimensional RC space onto the model signal. After adjusting the resulting *R* ^2^ value to account for the additional degrees of freedom in the regression, the resulting fit was significantly stronger than any of the three RCs alone—88% of the variability in the model signal was explained from the combined activity of the three RCs (Fig 5B).

## Discussion

The continuous flow of information between sensory and motor regions during decision-making has been previously inferred from a combination of behavioral and neurophysiological findings [5–9], and DV-like signals have been recorded over motor regions of the human brain using encephalography [11–14, 24]. Here we provide more direct neural evidence for the continuous flow paradigm of decision-making in human. By separating the task-evoked activity into distinct components, we showed that RT-predictive activity partitions into evidence-dependent (RC1) and evidence-independent components (RC2 and RC3). Importantly, the covariation of all components with RT was shown to be contemporaneous, and thus inconsistent with models containing sequential evidence-accumulation and motor output stages. Finally, a computational model strongly predictive of behavior on the decision task generated a DV best explained by the combined activity of all three derived neural components rather than any of their individual activities.

### Are the RCs truly distinct?

Due to the volume conduction inherent to EEG and the broadness of the RC topographies, one may wonder whether the RCs truly reflect distinct neural mechanisms. However, the uniqueness of the RC topographies, coupled with the linearity of the transfer between cortex and scalp, implies that the underlying cortical source distributions are different. In addition, the time courses of the three components exhibit distinct activation patterns (Fig 2B–2D). The differential behavior of the components with respect to the experimental variables is also not consistent with the components sharing a common neural substrate. For example, while RC1 shows a strong dependence on difficulty, RCs 2 and 3 do not (Fig 2B). While RC2 and RC3 are both independent of evidence, their distinctness is supported by the absence and presence of lateralization in RC2 and RC3, respectively (Fig 2A). Moreover, while RC2 shows covariation with RT after the button-press, RC3 does not (Fig 3A). Thus, the RCs represent three spatially and functionally distinct, contemporaneous covariates of RT.

### Interpreting the RCs

Due to its parietal topography and dependence on both strength-of-evidence and RT, we interpret RC1 as the classical evidence accumulation signal, analogous to that of area LIP in monkey [33]. Note, however, that the accumulated evidence signals for left and right motion are clearly mixed at the level of the scalp, as evidenced by RC1’s topography not varying with response-hand. Furthermore, RC1’s invariance to choice accuracy at time-of-response supports the idea that evidence accumulation terminates at a common fixed bound for both correct and incorrect decisions. It is also interesting to note that the amplitude of RC1 continued to increase right up until button-press, despite the fact that RC3 reached an inflection point approximately 100 ms before the response. We interpret this as the evidence accumulation process continuing for a short time after signaling commitment to the selected action (see interpretation of RC3 below).

Several of our findings support the idea that RCs 2 and 3 are dominated by sources in the premotor and motor cortex, respectively. Their activity covaries with RT but not task difficulty, while their topographies (frontocentral RC2 and contralateralized RC3) are consistent with premotor/motor origins. Moreover, RC2 and RC3 were shown to not vary with accuracy, as expected if their activity reflects planning and execution of the button-press. Another candidate mechanism which could explain covariation with RT but not difficulty is top-down attention; however, in this case, one would have expected such a source to vary with accuracy, which was not the case for any of the three RCs. The medial frontal topography of RC2 is consistent with a source in either the dorsal premotor cortex (PMd), which does not show contralateralized activity during tasks in which either hand may be used to signify a response [34–36], or the supplementary motor area, whose medial origin is unlikely to manifest in a lateralization on the scalp. The deviation between fast and slow responses after the button-press suggests that RC2 activity may also reflect decision uncertainty. Consistent with this notion, the role of the medial frontal cortex in performance monitoring has been extensively documented [37, 38]. The contralaterization of RC3 by response-hand, coupled with its invariance to RT following the button-press, implicates RC3 in action execution. It is thus tempting to speculate that a major contributor to RC3 is the primary motor cortex (M1). If activity in M1 indeed constitutes the bulk of RC3 activity, this suggests that variability in the behavioral manifestation of the decision process exists all the way until the final action execution, as evidenced by the late covariation of RC3 with RT (Figs 2B and 3A). Consistent with this notion, pre-movement variability in the firing rates of premotor and motor neurons has been found to predict RT on simple reaching tasks [39–41].

### Relation to previous work

**Fig 6:**
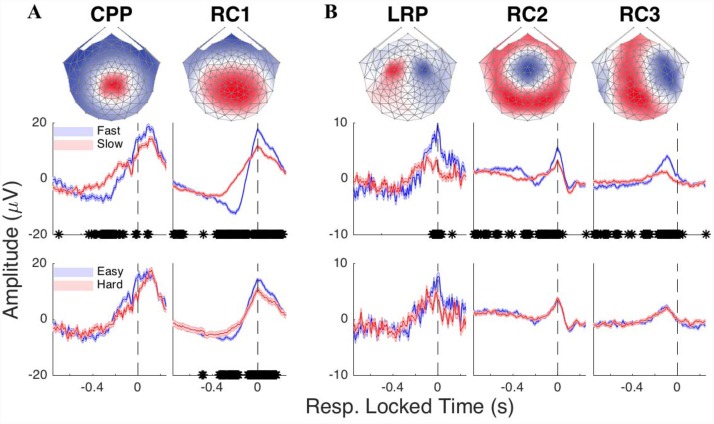
Contrasting the RCs with previously found decision-making signals. (**A**) Similar to RC1, the CPP exhibits a maximum over centroparietal electrodes and discriminates fast and slow responses leading up to the button-press. However, unlike RC1, the CPP peaks 100 ms after the response and is not modulated by difficulty. (**B**) The lateralized readiness potential (LRP) is odd-symmetric and concentrated over the lateral frontocentral electrodes, capturing only a subset of the broader RC3 topography. Moreover, compared to RC2 and RC3, the LRP is only briefly modulated by RT (shortly before the time-of-response). Put together, the use of trial-to-trial reliability in RCA identifies novel components (i.e., RC2) while capturing more covariation with the experimental variables than conventional, fixed components such as the CPP and LRP.

Recent human studies of decision-making have demonstrated attributes consistent with continuous flow. blackOur RC1 bears some similarity to the “centroparietal positivity” (CPP) identified by [12] and [24]. Fig 6A compares the scalp projections and time courses of the CPP with those of RC1. By construction, the CPP is marked by a focal positivity over the centroparietal electrodes, while its time course discriminates fast and slow trials akin to RC1. However, the CPP here peaks 100 ms after the button-press and does not discriminate difficulty. We attribute this difference to RC1 capturing a superset of the cortical generators, and thus experimental effects, of the CPP. The adaptive nature of RCA renders it flexible to detect sources of varying locations and orientations.

Another well-known EEG component previously shown to exhibit accumulation-like activity during perceptual decisions is the LRP [14, 24, 42], which has been recently suggested to threshold evidence accumulation in a three-layer model of perceptual decision-making [42–44]. We compared both RCs 2 and 3 to the LRP (Fig 6B), finding moderate correspondence between the LRP and RC3. The LRP captures some, but not all, of the broader RC3 topography, while its time course discriminates fast and slow responses, but only briefly near the time-of-response. Once again, this suggests that the LRP is picking up only a subset of the activity reflected by RC3.

Using a data-driven pattern classification approach, [45] recently identified a parietal component which discriminates difficulty and terminates at larger amplitudes for easy trials of a face-versus-car discrimination task, thus consistent with our RC1. We also mention that the choice-predictive activity identified by [11] over lateralized motor cortex was reproduced by our RC3 activity; namely, subtracting the activity of the two RC3s (left—right) led to a similar choice-predictivity leading up to the time of the button-press (data not shown). Moreover, our finding of RT-predictive activity at the onset of coherent motion is somewhat reminiscent of the recent report of pre-trial, choice-predictive oscillations over motor cortex [46]. The finding of a confidence-like signal in both RC1 and RC2 is consistent with recent results indicating that the probability of a correct choice is encoded by the same neural signals that index choice and RT [47]. In particular, high decision-confidence may be reflected in the greater slopes of RC1 and RC2 leading up to the button press. Note that in the case of RC1, a steeper gradient leading up to the behavioral response was coupled with steeper dynamics after the button press, adding support for a link between confidence and the rate of accumulation [48].

Our results argue for the merits of an adaptive component selection procedure such as RCA. By forming components in a data-driven manner, we captured more of the experimental variables (i.e., difficulty) and longer periods of covariation with RT. Importantly, these temporally extended periods of RT-discriminability across multiple components (only one of which is sensitive to task difficulty) provide strong evidence for the continuous flow model of perceptual decision-making. Finally, we uncovered a component which does not appear to have any analogues from previous work, namely RC2. This component may have been obscured in previous investigations due to the mixing of EEG sources at the scalp. Our use of trial-to-trial covariance allowed us to separate scalp signals into multiple components, one of which appears to be novel. Collectively, the recovered components brought together a multitude of disparate activation patterns within a unified multivariate framework linking neural activity to behavior.

### Neural basis of the decision variable

SSMs such as the DDM or OU model employed here are inherently univariate. Past attempts at linking neural activity to SSM predictions have focused on a single brain source at a time. At the core of these predictions is the DV, an abstract construct that has been very successful in predicting RT distributions and accuracy rates. However, the DV need not map onto a single brain region, and may span multiple distributed areas. We have shown that by incorporating the activity of multiple brain sources, a closer alignment between model prediction of behavior and neural activity was achieved, even after discounting for the additional degrees of freedom. This suggests that the behavioral DV maps onto multiple neural sources. One implication of our results is that the DV reflects a mixture of evidence accumulation and motor preparation, as RC2 and RC3 are evidence-independent signals that presumably reflect the planning and execution of the motor output. In other words, variability in the motor system shapes the DV. Historically, the contribution of the motor system to the DV has been in the form of an additive delay that reflects the time from commitment to action execution. The data presented here indicate that the effect of the motor system on the DV is more complex. Our results suggest that explicit modeling of concurrent evidence accumulation and motor planning is a critical but missing feature of SSMs, and that studies of the decision process will benefit from behaviorally informed multi-site recordings and appropriate multivariate techniques for linking brain to behavior.
